# The Appalachian Gap in Preventable Hospitalizations: Are We Seeing Any Progress?

**DOI:** 10.13023/jah.0502.02

**Published:** 2023-08-01

**Authors:** Rachel Hogg-Graham, Juan Lang, Teresa M. Waters

**Affiliations:** University of Kentucky, College of Public Health, rachel.hogg@uky.edu; University of Kentucky, College of Public Health; University of Kentucky

**Keywords:** Appalachia, preventable hospitalizations, rural health, longitudinal analysis

## Abstract

**Introduction:**

Previous studies have documented geographic variation in preventable hospitalizations between rural and urban areas, but much less is known about preventable hospitalization patterns between heterogeneous rural areas. Unique challenges related to access of care and poverty may put the rural Appalachian Region at risk for higher rates of preventable hospitalizations.

**Purpose:**

This study examines whether within-rural differences in Kentucky’s preventable hospitalization rates exist and how these differences may be changing over time.

**Methods:**

Longitudinal and geographic trends in county-level preventable hospitalization rates were examined using Kentucky hospital discharge data from 2016 to 2019. Regression models were run to determine whether changes over time in preventable hospitalization rates led to an increasing or decreasing gap in outcomes between rural Appalachian counties and their urban and rural non- Appalachian counterparts.

**Results:**

Rural Appalachian counties consistently had significantly higher preventable hospitalizations rates compared to their rural non-Appalachian and urban counterparts ( *p* < 0.01). A downward trend in overall preventable hospitalizations was observed for rural Appalachia over time, but trends were relatively stable for rural non-Appalachian and urban counties. Regression results indicate that there was no significant reduction in the “Appalachian gap” over time.

**Implications:**

The analyses confirm that rural areas within Kentucky experienced highly heterogeneous rates of preventable hospitalizations. Despite Medicaid expansion, there is little evidence of any narrowing of the “Appalachian gap.” Focus on improving access to care alone may be insufficient to improve outcomes. Alternative strategies that leverage population health approaches may improve capacity to address complex health and social needs in rural Appalachia.

## INTRODUCTION

Preventable hospitalizations are costly to our healthcare system and negatively impact individual quality of life.^1^ Considered an indicator of both healthcare quality and system efficiency, preventable hospitalizations are patient admissions that should rarely happen within a strong local healthcare system.^2^ Evidence also suggests that preventable hospitalizations can be attributed to lack of healthcare access and inadequate disease management.^2^ Not surprisingly, prior research has found higher preventable hospitalization rates in low-income, rural counties in the US.^3^,^4^

While previous studies have documented geographic variation in preventable hospitalizations between rural and urban areas, most research to-date has used a standard urban–rural subgrouping to examine differences.^3^,^5^ This approach treats rural areas as homogeneous and may not capture variation that exists within rural areas. Studies using this designation will group rural Appalachian, Delta, and frontier areas into the same categorization. In Kentucky, there are two relatively distinct types of rural areas: rural Appalachian and rural non- Appalachian counties. Compared to non-Appalachian areas, the Appalachian Region has faced persistently higher rates of poverty and limited access to care, potentially leading to higher rates of preventable hospitalizations.^6^ A 2014 study by Will et al. examining geographic variation in hypertension-related preventable hospitalizations among Medicare beneficiaries noted that counties with higher rates were found in the Appalachian Region.^5^

More research is needed to determine whether within-rural differences in preventable hospitalizations also exist for other conditions and populations and how these differences may be changing over time. To address this gap, this study examines geographic variation in four composite measures of preventable hospitalizations in Kentucky for the period 2016–2019. Kentucky contains diverse rural regions and ranks among the top five states with the highest rates of preventable hospitalizations.^7^

## METHODS

### Data Sources

Public use hospital discharge data for the state of Kentucky from 2016 to 2019 were obtained directly from the Kentucky Cabinet for Health and Family Services. The Agency for Healthcare Research and Quality’s (AHRQ) Quality Indicator (QI) software was used to calculate annual preventable hospitalization measures.^8^ Four composite measures, called prevention quality indicators (PQI), were calculated to capture all preventable hospitalizations, as well as subgroups of chronic, acute and diabetes-related hospitalizations (see Table S1 in this paper’s **additional files** section for a list of conditions in each PQI). In alignment with AHRQ’s methodology for examining preventable hospitalizations, PQIs were calculated at the county-level based on the patient’s county of residence. The data include all preventable hospitalizations at inpatient facilities for individuals aged 18 or over in Kentucky.

Counties were classified as urban, rural Appalachian, or rural non-Appalachian to examine geographic variation in preventable hospitalization rates. Counties were categorized as rural based on their –urban commuting area (RUCA) code. Although several rural classification systems exist, RUCA codes were selected to align with the Federal Office of Rural Health Policy’s definition. Using RUCA codes also allows for a more granular approach to how rural and urban communities are identified, helping to eliminate some of the homogeneity in a strict urban v. rural classification. Appalachian status was identified using the Appalachian Regional Commission’s listing of counties. Counties that were both urban and Appalachian were categorized as urban for the purpose of this analysis. The final Kentucky sample included four years of preventable hospitalization measures for 35 urban, 49 rural Appalachian, and 36 rural non- Appalachian counties.

### Statistical Analysis

A longitudinal ecological study was conducted to examine trends in preventable hospitalization rates across the Kentucky regions. Trends in all four composite preventable hospitalization measures were examined by geographic region using mean values of each PQI measure in each of the three regions. Values were weighted based on the population size of the county. Models were run for each PQI composite to determine whether changes over time (year) led to an increasing or decreasing gap in outcomes between rural Appalachian counties and their urban and rural non-Appalachian counterparts.^9^

## RESULTS

**Figure 1:**
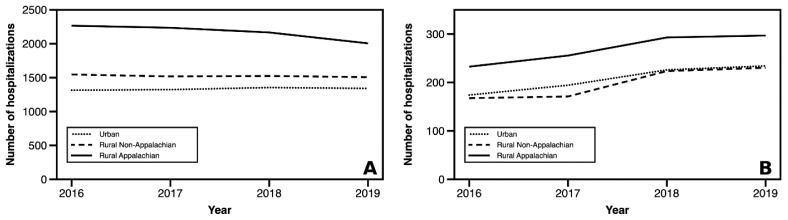
Longitudinal trends in all (A) and diabetes-related (B) trends in hospitalizations by geographic region, rate per 100,000 SOURCE: Authors’ analysis of KY hospital discharge data, 2016–2019 NOTE: Values weighted by county population size.

**Figure 2:**
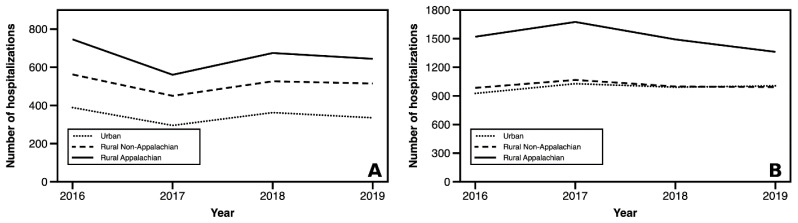
Longitudinal trends in acute (A) and chronic (B) preventable hospitalizations by geographic region, rate per 100,000 SOURCE: Authors’ analysis of KY hospital discharge data, 2016–2019 NOTE: Values weighted by county population size.

Rural Appalachian counties consistently had significantly higher preventable hospitalizations rates compared to their rural non-Appalachian and urban counterparts (Fig. 1 and Fig. 2, on next page). In 2019, rural Appalachian counties had 2,006 preventable hospitalizations per 100,000 members of their population. Rural non-Appalachian counties had the next highest rate at 1,507 hospitalizations per 100,000, while the rate for urban counties was 1,341 per 100,000. In addition, a downward trend in overall preventable hospitalizations for rural Appalachian counties over time was observed, but relatively stable trends for rural non-Appalachian and urban counties were seen. These disparate trends resulted in a modest narrowing of the “Appalachian gap” (rural Appalachian v. rural non-Appalachian) by 221 preventable hospitalizations per 100,000.

Consistent patterns were found in an Appalachian gap across all PQIs (Fig. 1 and Fig. 2). The discrepancies between rural non-Appalachian and urban counties were typically small, and in the case of diabetes-related hospitalizations, almost non-existent (Fig. 1B). Trends in the acute and chronic hospitalization subgroups varied over time (Fig. 2), with modest closing of the gap between rural Appalachian and rural non-Appalachian. Urban communities experienced a small increase in chronic hospitalizations from 2018 to 2019.

Results from the regression models confirmed trends observed in the longitudinal figures and provide insight on the statistical significance of changes over time. Specifically, results (Table 1, p. 11) indicated that, compared to Appalachian counties, urban and rural non-Appalachian preventable hospitalization rates were significantly lower (*p* < 0.01). Results also confirmed that overall preventable hospitalizations and acute preventable hospitalizations were falling over time (*p* < 0.10), diabetes-related preventable hospitalizations were increasing over time (*p* < 0.01) and chronic preventable hospitalizations did not change significantly over time. Finally, results suggest that there was no significant reduction in the Appalachian gap over time.

## DISCUSSION

Analyses of Kentucky hospital discharge data for 2016–2019 confirm that rural areas within Kentucky experience highly heterogeneous rates of preventable hospitalizations. Specifically, there is significant evidence of an “Appalachian gap” between rural Appalachian and rural non-Appalachian counties. Rural non-Appalachian counties have outcomes far closer to urban Kentucky counties, apart from acute preventable hospitalizations. While the remoteness of Appalachian counties, along with their history of poverty, make this result somewhat intuitive, it is unclear how much attention additional inequities in the region have garnered among policymakers. In light of these findings, policies and programs that target rural areas may need additional tailoring to ameliorate the “Appalachian gap.” For example, efforts to enhance broadband connectivity in rural areas have experienced some success in improving telehealth access; yet they face significant logistical barriers in rural, mountainous areas. Similarly, transportation challenges in accessing health and social services may be particularly challenging because of the geographic landscape. Addressing the extreme poverty and associated unmet social needs in Kentucky’s rural Appalachian counties may be essential to seeing any real progress in health outcomes. Additional research is needed to determine whether the identified in this study also exists in other states with heterogeneous rural areas.

These analyses also highlight reductions in overall preventable hospitalizations and acute preventable hospitalizations over time (*p* < 0.10). Since Kentucky is a Medicaid expansion state, these results are consistent with previous work that found Medicaid expansion was associated with reductions in a variety of PQI measures across 36 states between 2009 and 2015.^10^ Of concern, findings indicate a significant increase in diabetes-related preventable hospitalizations, suggesting that improvements may not be sustained or universal. A longer panel with additional states is needed to confirm whether patterns among diabetics deserve special attention.

Very little evidence of any narrowing of the “Appalachian Gap” between 2016 and 2019 was found, despite the enhanced access to care associated with 2014 Medicaid expansion in Kentucky. While this is disappointing, other research on the impact of the Affordable Care Act (ACA) and Medicaid Expansion has made similar findings. That is, while ACA and Medicaid expansion have been associated with health improvements across population groups, the disparities in outcomes between resourced and underserved groups have not necessarily narrowed.^11^ Again, the results suggest that more tailored approaches may be needed to close the gap between Appalachian and non-Appalachian areas.

## IMPLICATIONS

As the results indicate, more tailored approaches could yield meaningful improvements in preventable hospitalizations in rural Appalachia. Emerging evidence suggests several possibilities, including investing in public health and social service systems and supporting multisector linkages to better address social determinants of health.^12^ Traditional strategies that focus on strengthening clinical care systems to improve outcomes may not be sufficient to reduce the persistently high preventable hospitalization rates in rural Appalachia. Further, rural hospitals that experience resource challenges may gain financially from preventable hospitalizations, despite the cost to the patient. Considering alternative strategies that use a population health approach to improve the region’s capacity to address complex health and social needs may be warranted. These approaches are likely to require significant investment and local flexibility to generate meaningful improvements, but that investment could also be used to support other value-based initiatives in rural areas. The collaborative nature of population health approaches will also require initiatives to be multisector with a focus on strengthening primary care, public health, and social care in communities. Policymakers should consider ways they can meaningfully support and encourage the building of strong population health systems in rural regions as a mechanism to reduce the rate of costly preventable hospitalizations.

**SUMMARY BOX**

**What is already known about this topic?**

Preventable hospitalizations are costly, common and have been attributed to lack of healthcare access and inadequate disease management. Prior research has also documented higher preventable hospitalizations in low-income, rural U.S. counties.

**What is added by this report?**

This report explores whether there are significant differences in preventable hospitalizations between heterogenous rural areas. In Kentucky, rural Appalachian counties may be at particular risk for preventable hospitalizations because of unique problems with access to care and extreme poverty.

**What are the implications for future research?**

Findings show a significant “Appalachian gap” in preventable hospitalization rates and little evidence of any narrowing of this gap over time (2016–2019). More tailored approaches may be needed to close the gap in health outcomes between rural Appalachian and rural non-Appalachian areas.

## Supplementary Information



## Figures and Tables

**Table 1:** Results from longitudinal regression models comparing changes in KY preventable hospitalization rates by geographic region, 2016–2019

	Model 1	Model 2	Model 3	Model 4
	All	Acute	Chronic	Diabetes-related
Urban	−0.0118 †	−0.0046 †	−0.0072 †	−0.0008 †
Rural non-Appalachia	−0.0032 †	−0.0027 †	−0.0055 †	−0.0006 †
Time	−0.0028*	−0.0013*	−0.0015	0.0007 †
Urban*time	0.0034	0.0008	0.0026	−0.0001
Rural non-Appalachia*time	0.0023	0.0008	0.0014	−0.0002

NOTES: Rural Appalachian counties serve as the reference category. Symbols indicate significance levels:

* *p* < 0.10

† *p* < 0.01
