# Supplementary material for: The Appalachian Gap in Preventable Hospitalizations: Are We Seeing Any Progress?
Source: J Appalach Health. 2023 Aug 1;5(2):5–14. doi: 10.13023/jah.0502.02 (PMC10629885; doi:10.13023/jah.0502.02)
Supplement: Supplementary file 1 [file 5.2.2_Hogg-GrahamandLang_AdditionalFile.pdf]

Table S1. Hospitalizations included in Prevention Quality Indicators

**Chronic**
Diabetes short-term complications
Diabetes long-term complications
COPD or asthma in older adults
Hypertension
Heart failure
Uncontrolled diabetes
Asthma in younger adults
Lower extremity amputation among patients with diabetes
**Acute**
Community-acquired pneumonia
UTI
**Diabetes-related**
Diabetes short-term complications
Diabetes long-term complications
Uncontrolled diabetes
Lower extremity amputation among patients with diabetes

Notes. Measure of all preventable hospitalizations includes all conditions
